# Supplementary material for: Mechanisms governing the pioneering and redistribution capabilities of the non-classical pioneer PU.1
Source: Nat Commun. 2020 Jan 21;11:402. doi: 10.1038/s41467-019-13960-2 (PMC6972792; doi:10.1038/s41467-019-13960-2)
Supplement: Supplementary file 7 — Source data [file 41467_2019_13960_MOESM7_ESM.zip › Source_Data/Figure6/Figure6B_MotifScanOutput/ETS1_PU.1/homerResults/motif23.similar.html]

motif23

## Information for motif23

C
T
G
A
T
C
G
A
A
G
T
C
A
G
T
C
C
G
T
A
A
G
T
C
C
G
T
A
C
G
A
T
T
A
G
C
C
G
T
A
T
C
G
A
C
G
T
A
T
C
A
G
T
G
C
A
  
Reverse Opposite:  

A
C
G
T
A
G
T
C
A
C
G
T
A
G
C
T
A
C
G
T
A
T
C
G
G
C
T
A
C
G
A
T
A
C
T
G
A
C
G
T
A
C
T
G
A
C
T
G
A
G
C
T
G
A
C
T
  

|  |  |
| --- | --- |
| p-value: | 1e-101 |
| log p-value: | -2.343e+02 |
| Information Content per bp: | 1.748 |
| Number of Target Sequences with motif | 669.0 |
| Percentage of Target Sequences with motif | 4.23% |
| Number of Background Sequences with motif | 350.8 |
| Percentage of Background Sequences with motif | 1.09% |
| Average Position of motif in Targets | 102.1 +/- 45.6bp |
| Average Position of motif in Background | 100.8 +/- 67.5bp |
| Strand Bias (log2 ratio + to - strand density) | 0.0 |
| Multiplicity (# of sites on avg that occur together) | 1.01 |
| Motif File: | file (matrix) reverse opposite |

### Similar de novo motifs found

|  |  |  |  |  |  |  |  |
| --- | --- | --- | --- | --- | --- | --- | --- |
| Rank | Match Score | Redundant Motif | P-value | log P-value | % of Targets | % of Background | Motif file |
| 1 | 0.777 | G T A C C G T A T A C G G C T A C G A T T G A C C G T A T C G A C G T A C T A G | 1e-99 | -228.465912 | 11.00% | 5.49% | motif file (matrix) |
| 2 | 0.800 | T A G C C G A T G A C T C G A T A T C G C G T A C G A T A T C G A C G T | 1e-94 | -218.353129 | 13.47% | 7.44% | motif file (matrix) |
| 3 | 0.844 | T G A C G C T A A T G C G C T A C A G T T A G C G C T A T G C A C T G A T C A G T G A C | 1e-83 | -191.991663 | 8.86% | 4.32% | motif file (matrix) |
